# Acculturative risk or selective retention? A longitudinal study of binge drinking in Asian and Latinx immigrant youth

**DOI:** 10.1016/j.jmh.2026.100415

**Published:** 2026-05-15

**Authors:** Zobayer Ahmmad

**Affiliations:** Department of Sociology, University of Vermont, Burlington, VT, USA

**Keywords:** Alcohol consumption, Acculturation, Selective acculturation, Immigrant health, Racial/ethnic disparities, Longitudinal study

## Abstract

Alcohol consumption is a significant public health concern in the US, increasingly impacting racial/ethnic minority youth. This study investigates binge drinking from adolescence to mature adulthood among Asian and Latinx Americans, using three waves of Add Health data and mixed-effects (melogit) modeling technique. Nativity and gender were used as key moderating variables. Guided by the acculturative risk framework, which posits that immigrants' healthier behavioral profiles diminish with longer US residence, the findings from this study offer only partial support for this hypothesis but provide stronger evidence for selection effects. Immigrants reported lower binge drinking rates than their US-born counterparts. While some acculturative risk was observed (e.g., English use among Latinx individuals), most immigrant segments maintained healthier profiles. Notably, immigrant females, particularly within the Latinx group, exhibited the flattest binge drinking trajectories, highlighting gender as a fundamental indicator of selective retention of alcohol consumption among US immigrants.

## 1 Introduction

Alcohol remains a significant public health problem among U.S. youth, with substantial disparities across racial and ethnic groups. According to the CDC, alcohol is the most common substance used by individuals under 21 (22 percent vs. 4 percent for cigarettes) and becoming more frequently used substance for girls (Hoots, 2023). Although drinking among young adults (18–34) has declined since the early 2000s, this decline is weaker among racial/ethnic minority youth (10.9 percent vs. 13.1 percent) between 2001 and 2023 (Gallup, 2023). Excessive alcohol consumption results in approximately 4000 youth deaths annually and imposes substantial economic costs (Centers for Disease Control and Prevention, n.d.). Health consequences include increased risk of chronic diseases (e.g., diabetes, cardiovascular conditions, cancer) and, among adolescents, poorer academic performance, violence, delinquency, and impaired cognitive and mental health (Bacio et al., 2013; Bagnardi et al., 2015; Greene and Maggs, 2018; Maldonado-Molina et al., 2011; Sowell et al., 1999).

Demographic shifts have amplified the public health relevance of studying alcohol consumption among race/ethnic minority youths. The Hispanic population reached 62.1 million in 2020 (18.9 percent of the U.S. population), with youth comprising over 25 percent of this group and one in three Hispanics being immigrants (Lopez and Krogstad, 2022; Ruiz et al., 2021). Similarly, Asian American youth represent 28 percent of their population, with rapid growth (33 percent from 2010 to 2020) and over half being foreign-born (Budiman et al., 2024; Ruiz et al., 2021). Consequently, the proportion of youth drinkers from racial/ethnic minorities rose from 28 percent to 50 percent between 2001 and 2023 (Gallup, 2023). NSDUH data show past-month alcohol use among Hispanic youth ages 12–17 at 8.6 percent, rising sharply to 42.4 percent among those 18–25 (Substance Abuse and Mental Health Services Administration, 2022). Asian adolescents report low initial use (3.9 percent) that increases to 32.2 percent in early adulthood, though overall consumption remains lower than among Whites (38 percent vs. 59.8 percent) (SAMHSA, 2022).

Research has highlighted acculturative stress, nativity, generational status, and social networks as key factors influencing alcohol use (Cook et al., 2013; Lui and Zamboanga, 2018; Park et al., 2014; Wahl and Eitle, 2010; Zemore, 2007). Numerous studies document an "immigrant health advantage" in alcohol consumption patterns upon arrival in the U.S. However, consistent with acculturative stress theory, this protective effect often diminishes over time due to acculturative pressures (Alamilla et al., 2020; Eitle et al., 2009; Greene and Maggs, 2018; Park et al., 2014; Salas-Wright et al., 2014). However, emerging evidence indicates heterogeneity in these risks by age, ethnicity, and gender (Ahmmad et al., 2023). To clarify these mechanisms, this study applies acculturative risk and selective acculturation frameworks to longitudinal data from Add Health, examining alcohol use trajectories among Asian and Latinx youth, with emphasis on gender and nativity. Findings aim to support tailored interventions for these growing demographic groups.

## 2 Literature review

### 2.1 Lower risks of alcohol consumption among immigrants

Studies find that immigrant youth across various ethnicities generally exhibit lower alcohol consumption compared to their U.S.-born counterparts (Alamilla et al., 2020; Bacio et al., 2013; Eitle et al., 2009). Specifically, studies using the Add Health data find that immigrant adolescents from Mexican, Cuban, and Puerto Rican origins have lower alcohol consumption, binge drinking, or alcohol-related problems than their U.S.-born peers (Bacio et al., 2013; Eitle et al., 2009; Kopak, 2013; Peña et al., 2008). Another study conducted using the University Life Study found that foreign-born students of Latinx and Asian origins had lower alcohol use and less intention to get drunk than their U.S.-born peers (Greene and Maggs, 2018). Alamilla et al. (2020) also used college student samples from multiple settings and found lower alcohol consumption among foreign-born students compared to their U.S.-born peers (Alamilla et al., 2020).

A lower risk of alcohol consumption among immigrants has also been found in other countries. A study in Sweden, utilizing data from multiple sources including the Total Population Census, finds that immigrant youth ages 15 and older from >64 countries had lower alcohol use disorders between 2010 and 2015 (Cook et al., 2013). In Norway, a reported decline in youth alcohol consumption since 1996 has been attributed to an increasing proportion of youth who are immigrating from non-drinking cultures (Rogne et al., 2019). Furthermore, Barsties et al. (2017) analyzed data from the 2013/14 Health and Behavior in School-aged Children (HBSC) study, a school-based survey conducted by the World Health Organization (WHO) across 23 countries, with immigrant participants from over 130 countries. Their findings show that immigrant adolescents report lower lifetime alcohol use compared to their native-born peers (Barsties et al., 2017).

Given the protective effect found on various measures of alcohol consumption among immigrants regardless of ethnic background or recipient countries, I hypothesize that: Immigrant adolescents will exhibit lower alcohol consumption compared to their native-born peers, regardless of ethnicity (Hypothesis 1).

### 2.2 The unfolding paradox: Acculturation and increased risks of alcohol consumption

Despite an initial advantage in alcohol consumption among immigrant youth, the risk of use and related problems rapidly increases as they spend more years in the US (Lui and Zamboanga, 2018; Zemore, 2007). This has been a concept originally hypothesized in Milton Gordon's linear assimilation paradigm, which suggests that immigrants gradually adopt native cultures and languages, potentially leading to increased alcohol consumption (Schwartz et al., 2010). This idea is supported by several studies that use length of stay as an indicator of acculturation, showing a positive association with alcohol consumption (Park et al., 2014). For example, Park et al. (2014) used data from the National Latino and Asian American Study (NLAAS) to show that length of residence in the US is associated with both the frequency and quantity of alcohol consumption among various Asian ethnicities (Park et al., 2014). Furthermore, Kopak (2013) analyzed multiple waves of the Add Health data and, using latent growth curve modeling, finds that while immigrants have lower alcohol consumption at the baseline (adolescence), the rate of increase is steepest among them compared to second-generation immigrants (γ=0.06 vs. 0.04).

Additional support for the linear assimilation framework comes from findings based on generational differences in immigrant alcohol consumption (Ahmmad and Adkins, 2021; Wahl and Eitle, 2010). Several studies show linear increases in the levels and rates of alcohol consumption across immigrant generations, defined as first-generation (immigrants), second-generation (children of immigrants), and third or later generation (grandchildren of immigrants) (Ahmmad and Adkins, 2021; Schwartz et al., 2010; Wahl and Eitle, 2010). Among Mexican and Puerto Rican youth from the Add Health study, there have been linear increases in alcohol use and binge drinking across different generations (Wahl and Eitle, 2010). Ahmmad and Adkins (2021), analyzing multiple waves of the Add Health data, also find a similar generational increase in regular alcohol use (≥1–2 days per week) among various Asian ethnicities.

Researchers also use other indicators of acculturation to assess the risks of greater alcohol consumption among immigrant youth. One of the most common indicators is language use of the host culture, particularly English in daily conversations (Ahmmad and Adkins, 2021; Bryant and Kim, 2013; Lui and Zamboanga, 2018; Park et al., 2014; Zemore, 2007). Since language acquisition takes time, many argue that a positive association between English use and alcohol consumption supports the linear assimilation framework (Schwartz et al., 2010). For instance, a study using the NLAAS data finds that English proficiency in reading, writing, and speaking is associated with increased alcohol consumption among Asian adults (Park et al., 2014). English use in intimate settings has also been found to be associated with increased alcohol consumption among Asian youth in the Add Health study (Ahmmad and Adkins, 2021).

Besides language use, a greater degree of acculturation can be assessed through various scales developed over the past few decades (Lui and Zamboanga, 2018; Zemore, 2007). These scales often focus on behavioral and value-oriented indicators of acculturation. For example, factors such as hanging out with native friends or friends from other ethnicities, idealizing American norms and values, and supporting individualism are often included in these acculturation scales. A meta-analysis based on 31 studies published since 1979 finds that acculturative factors are positively associated with alcohol consumption and the intensity of hazardous drinking among Asians (Lui and Zamboanga, 2018). Additionally, Zemore (2007) reviews 32 studies involving Latino adult samples and finds that acculturation has been associated with volume and frequency of alcohol consumption (Zemore, 2007).

In summary, the initial protective effect against alcohol consumption observed among immigrant youth is not permanent. The cumulative evidence supports that a longer length of stay, generational progression, and greater English language proficiency are linked to higher rates of alcohol use. The line of findings suggests a clear trend: the initial protective factor associated with being a recent immigrant diminishes over time supporting Gordon’s linear assimilation paradigm. Therefore, I hypothesize that as immigrant youth transition into adulthood, their initial protective advantages in alcohol use will diminish, leading to increased consumption rates that converge with those of their native-born peers (Hypothesis 2).

### 2.3 The protective power of selection: Weakened acculturation risks for immigrant alcohol use

Selective acculturation posits that retaining aspects of one's heritage culture can balance out the negative effects of acculturative risks (Schwartz et al., 2010). This has also been considered a strategy for individual efforts to maintain better health behaviors when facing the risk of losing them through complete assimilation (Ahmmad et al., 2023; Schwartz et al., 2010). This idea is grounded in the expansive paradigm of Gordon's straight-line assimilation framework proposed by John Berry, which moves beyond a simple, linear acquisition of a host culture. Instead, Berry (1980) model focuses on a multidimensional aspect of assimilation that includes the retention of one's heritage culture (Berry, 2017; Schwartz et al., 2010). The different degrees of interaction between these two orientations create four distinct paths of assimilation: 1) assimilation (adopting the host culture and rejecting the heritage culture), 2) separation (rejecting the host culture but retaining the heritage culture), 3) integration (adopting the host culture while retaining the heritage culture), and 4) marginalization (rejecting both the heritage and host cultures) (Schwartz et al., 2010).

Berry's multidimensional acculturation model has been receiving increased attention from researchers, including Portes and Zhou, whose work on segmented assimilation taps into the recent influx of immigrants from around the world, creating a complex landscape of assimilation to US society (Jasso et al., 2004). Since contemporary immigrants vary considerably in socioeconomic, religious, and cultural aspects, their context of reception and subsequent acculturation follows some parallels to their pre-migration contexts, and different paths of assimilation are expected (Schwartz et al., 2010). In fact, much research is now producing evidence that not all immigrant assimilation translates into negative health outcomes. Instead, adopting a bicultural strategy to assimilation has been associated with the most favorable psychosocial and health behaviors (Ahmmad et al., 2023; Berry, 2017; Sall, 2020; Schwartz et al., 2010; Yeh et al., 2009).

The current literature that links acculturation indicators like length of stay and English use to health behaviors, including alcohol consumption, relies heavily on cross-sectional data (Bacio et al., 2013; Eitle et al., 2009; Kopak, 2013; Lui and Zamboanga, 2018; Peña et al., 2008; Zemore, 2007). Using length of stay as the sole predictor of immigrant adaptation over time is questionable, as it represents a linear perspective that oversimplifies the experience of contemporary immigrants. This approach ignores complexities such as transnational communication, experiences with discrimination, or a lack of desire to integrate into the host society (Ahmmad et al., 2023). Similar limitations are present with using English proficiency as an indicator of assimilation; it can be an unreliable measure, as a person's initial English skills may be high due to factors like skilled migration from English-speaking countries or those with a history of British colonization, such as India (Ahmmad et al., 2023; Schwartz et al., 2010). Furthermore, many acculturation scales assess only one dimension of the process, which also reinforces the flawed straight-line assimilation perspective (Lui and Zamboanga, 2018).

A focus on biculturalism or selection, however, addresses some of the challenges with the straight-line assimilation theory. Many researchers are starting to consider ethnicity as a powerful indicator of selection (Nagasawa et al., 2001). As immigrants come from diverse origins, from wealthy nations like Japan and China to poorer ones such as Mexico and Bangladesh, a person’s ethnicity can signal specific aspects of their selection (Esser, 2004; Melville, 1980). People from some ethnic origins can buffer the negative effects of learning English or having friends from the host society through strong community resources or familial connections. Others, however, may be forced into unhealthy environments due to poorer neighborhoods or inner-city locations, and their acculturation may translate into poorer health behaviors due to a lack of community or ethnic buffers (Esser, 2004). For instance, one study finds that maintaining co-ethnic friend networks is associated with reduced alcohol consumption among Mexican adolescents but not among Cubans and Puerto Ricans (Eitle et al., 2009).

One notable gap in acculturation research on alcohol use is the role of gender. Like ethnicity, gender can also serve as a buffer against the negative effects of acculturation (Ahmmad et al., 2023; Lui and Zamboanga, 2018; Wahl and Eitle, 2010; Yeh et al., 2009). This is a particularly interesting area to explore, as many developing nations have strong gender norms regarding behaviors like smoking and drinking, with more relaxed attitudes for boys and stricter expectations for girls (Ahmmad et al., 2023; Kamimura et al., 2018). Whether these norms translate into the American acculturation process remains a core question. Indeed, one meta-analysis on 31 studies of alcohol consumption among Asians finds that gender is a significant modifier in the analysis conducted using mixed-effects modeling techniques (Lui and Zamboanga, 2018). Similarly, gender has been found to be a strong moderator of alcohol use and binge drinking among Latinx adolescents from the Add Health study (Wahl and Eitle, 2010).

A recent longitudinal study by Ahmmad et al. (2023) examined gender's role in the acculturation of Asian immigrant youth, using regular smoking as a culturally sensitive health behavior. The findings reveal that immigrant girls maintain very low smoking rates compared to both their U.S.-born and male counterparts, despite having similar educational and language acquisition outcomes over time. This suggests a powerful gender-based selection in smoking. In a related, ongoing study presented at the Population Association of America 2025 conference, Ahmmad expanded the analysis to include Latinx youth, using body mass index (BMI) as a less-stigmatized health indicator. This research indicates that the protective effect of gender is also influenced by a person's ethnicity and their initial level of selection upon migration.

The concept of acculturation, as a multidimensional process rather than a linear one, challenges the idea that immigrant youth's health behaviors simply converge with those of their native-born peers over time. While studies show an increase in alcohol consumption with a longer stay, generational progression, and greater English use, these are often based on cross-sectional data that ignore unfolding differences across different immigrant groups and across gender. Based on the evidence above that the protective effect is not uniform and may vary significantly by gender and ethnicity, I hypothesize that as immigrant youth transition into adulthood, immigrant females will show a weaker convergence toward native-born peers' alcohol use rates than immigrant males, and that this gender-based selection will vary by Asian and Latinx ethnicity (Hypothesis 3).

### 2.4 Data and methods

#### 2.4.1 Data

The data for this project comes from the National Longitudinal Study of Adolescent to Adult Health (Add Health) (Harris et al., 2019). A general description of the study can be found in Ahmmad and Adkins (2021). Add Health is a panel study that began collecting information from over 20,000 adolescents in grades 7–12 between 1994–95. Despite some participant attrition, it is an ongoing study that has completed five Waves of data collection, with subsequent Waves collected in 1996 (Wave 2), 2001–02 (Wave 3), 2008–09 (Wave 4), and 2016–18 (Wave 5). The study has been collecting various types of information from respondents, including their demographics, family backgrounds, behaviors, and health. A large volume of studies has been published using the Add Health data, which is available in both open and restricted access formats.

#### 2.4.2 Analytical sample

The overall analytical sample consists of 3767 respondents (with non-missing information) who self-identify as either Latinx or Asian in Wave 1. The total sample size decreases over time to 3449 in Wave 3 and 2187 in Wave 5. The number of Latinx respondents with non-missing information is 2701 in Wave 1, 2263 in Wave 3, and 1475 in Wave 5. The sample sizes for Asian respondents are 1066 in Wave 1, 1186 in Wave 3, and 712 in Wave 5. The slight increase in the Asian subsample in Wave 3 is primarily due to oversampling including more respondents from the original Wave 1 school-based surveys (Harris et al., 2019). The analytical sample included 9404 respondents (2965 Asians and 6439 Latinx) (organized long) from Waves 1, 3, and 5.

#### 2.4.3 Outcome variable

The dependent variable for this analysis is binge drinking, consistent with prior literature (Bacio et al., 2013; Kopak, 2013). In Waves 1 and 3, respondents were asked: "During the past 12 months, on how many days did you drink *five or more drinks in a row*?" The full scale included the following categories: 1=every day or almost every day, 2 = 3–5 days per week, 3 = 1–2 days per week, 4 = 2–3 days per month, 5=once a month or less (3–12 months in past 12 months), 6 = 1–2 days in past 12 months, and 7=never. Respondents who selected any option from 1 to 6 were classified as having engaged in binge drinking (coded as 1), while those who selected option 7 were classified as having never engaged in binge drinking (coded as 0).

In Wave 5, the question was updated to reflect gender-specific binge drinking thresholds (Wilsnack et al., 2018). Respondents were asked how often they had "4 [if female] or 5 [if male] or more drinks in a row in the past 12 months?" The full scale included the following categories: 0=none, 1 = 1 or 2 days in the past 12 months, 2=once a month or less (3 to 12 days in the past 12 months), 3 = 2–3 days a month, 4 = 1 or 2 days a week, 5 = 3 to 5 days a week, 6=every day or almost every day. Respondents who selected any option from 1 to 6 were classified as having engaged in binge drinking (coded as 1), while those who selected option 0 were classified as having never engaged in binge drinking.

For all waves, legitimate skips (originally coded as 97) were recoded to 0 in the final binary variable.

#### 2.4.4 Independent variables

Information on the respondents' ethnicity was derived from Wave 1. Respondents were asked to self-identify their race as one of five categories: White, Black or African American, American Indian or Native American, Asian or Pacific Islander, or Other. To further refine the race/ethnicity reports, separate questions were used to identify specific Asian origins (Chinese, Filipino, Japanese, Asian Indian, Korean, Vietnamese, or Other Asian) and Hispanic or Latino origins. These detailed questions allowed us to parse out individuals of Asian or Hispanic/Latino descent from those who may have selected multiple or broader racial categories, ensuring a more accurate Asian or Latinx classification.

Gender and nativity were measured using the respondents’ self-reported sex and nation of origin. Four primary categories were created for the overall sample, including immigrant male, immigrant female, native-born male, and native-born female. These categories were further stratified by ethnic identity to create a total of eight groups: 1) Asian immigrant male, 2) Asian immigrant female, 3) Asian native-born male, 4) Asian native-born female, 5) Latinx immigrant male, 6) Latinx immigrant female, 7) Latinx native-born male, and 8) Latinx native-born female.

Self-reported age variable was included as a continuous variable. The acculturation variable, use of English in personal conversation, is available in Waves 1 to 3. There was relatively little within-participant variation in English use across Waves; therefore, on the basis of the English use information from Waves 1 to 3, this measure was estimated for Wave 5 as the average of preceding Waves. The English use variable was dichotomized, with 1 indicating English use and 0 indicating no use of English, in personal conversation. To construct co-ethnic friend network proportion measure, Wave 1 in-school friendship nomination data were used. Each respondent was allowed to nominate up to 10 friends. The friend nominations were matched with students of known race in the in-school sample enabling calculation of co-ethnic friendship proportion as the number of known Asian/Latino friends divided by the total number of friends with known race (Asian co-ethnic=#known-Asian friends/total # known-race friends; Latino co-ethnic=#known-Latino friends/total # known-race friends).

The measures of education were assessed across multiple Waves. In Wave 1, education represented the parents' education level, whereas in Waves 3 and 4, it was derived from the respondents' reports on their highest education level achieved. Education level is a dichotomous variable where a value of 0 represents high school graduation and below, and a value of 1 represents some college and above.

#### 2.4.5 Analytical technique

Descriptive statistics, including means, percentages, and standard deviations (SD), were calculated for the combined sample (organized long) and for each group individually. Multilevel mixed-effects logistic regression (melogit) models were used to derive inferential estimates for assessing the effects of selective acculturation on the odds of alcohol consumption. Mixed effects modeling technique offers advantages over traditional logistic regression because it handles the hierarchical structure of the data, where individual observations are clustered across Waves (Johnson et al., 2013). By incorporating random effects, the models accounted for the non-independence of observations within the same cluster. The analysis included a series of melogit models on the overall sample. These models progressively built upon each other by adding fixed-effect covariates such as age, wave, gender, education level, nativity, ethnicity, English use, and co-ethnic friend networks as well as cross-classifications of nativity, gender, and ethnicity. To test for non-linearity, a model considering a linear age term was compared to a model incorporating both linear and quadratic terms (age squared). A likelihood-ratio test indicated that the quadratic model significantly improved the model fit, χ²(1) = 82.24, p < 0.001. Models 5 to 7 were used to determine the interactive effects nativity, gender, and ethnicity on the odds of alcohol consumption. Sensitivity analyses were conducted to check the robustness of the findings, primarily investigating the interactive effects of nativity, gender, and English use.

## 3 Results

Table 1 presents descriptive statistics for the overall analytic sample (N = 9404) and stratified by ethnicity (Asian, n = 2965; Latinx, n = 6439) and further disaggregated by nativity and gender. The sample statistics are based on the variables measured across Waves 1, 3, and 5.

**Table 1:** Descriptive statistics of sample (Organized Long) Stratified by U.S. Asian and Latinx ethnicity, the national longitudinal study of adolescent to adult health 1994–2018, waves 1, 3, & 5 (Obs. =9404).

Overall (N = 9404)						Asian (n = 2965)				Latinx (n = 6439)			
						Im. Women	Im. Men	US-born Women	US-born Men	Im. Women	Im. Men	US-born Women	US-born Men
Variable	Mean/%	SD	Min	Max		Mean/%	Mean/%	Mean/%	Mean/%	Mean/%	Mean/%	Mean/%	Mean/%
Alcohol consumed (WI, III, V)	35%	-	0	1		20%	31%	33%	40%	19%	36%	34%	45%
Female	50%	-	0	1		-	-	-	-	-	-	-	-
Foreign-born	29%	-	0	1		-	-	-	-	-	-	-	-
Age (WI, III, V)	23.47	8.28	12	42		24.21	24.13	24.19	23.08	23.84	23.17	23.70	22.75
Education (WI,III,V)	54%	-	0	1		77%	75%	74%	72%	44%	37%	49%	43%
English use in intimate settings (WI, III, V)	60%	-	0	1		53%	53%	87%	84%	19%	17%	66%	67%
Co-ethnic Friend Network (Asian)^+^	14%	-	0	1		45%	40%	36%	37%	1%	1%	2%	3%
Co-ethnic Friend Network (Latino)^+^	27%	-	0	1		7%	7%	13%	10%	46%	36%	37%	29%

Abbreviations: Im=Immigrant; + Indicates proportion co-ethnic friends.

The overall sample had 29 percent foreign-born respondents, with a mean age of 23.4 years (SD=8.2). Out of the total respondents, thirty-five percent of the total sample reported alcohol consumption across the waves. A majority (54 percent) reported having some college or above level of education, and 60 percent reported primarily English use in intimate settings. The proportion of co-ethnic friends was notably higher among Latino respondents than Asian respondents overall (27%vs. 14%). Across both groups, immigrant respondents were more likely than their US-born counterparts to report having predominantly co-ethnic friendship networks.

Notable disparities emerge when the sample is stratified by ethnicity, nativity, and gender. Asian respondents exhibited lower overall prevalence of alcohol consumption (20–40 percent) compared to the total sample, with the lowest rate among foreign-born Asian women (20 percent) and the highest among U.S.-born Asian men (40 percent). This subgroup also showed the highest educational attainment (72–77 percent) and a substantial difference in language use across nativity: while a majority of U.S.-born Asians used English in intimate settings (84–87 percent), only about half (53 percent) of their foreign-born counterparts did so.

Latinx respondents showed a wider range in alcohol consumption (19–45 percent), with foreign-born Latinx women reporting the lowest rate (19 percent) and U.S.-born Latinx men the highest (45 percent). Educational attainment was substantially lower for Latinx respondents (37–49 percent) compared to Asian respondents. A similar, though more extreme, nativity gradient was observed for language use. The vast majority of U.S.-born Latinx individuals reported English use (66–67 percent), compared to a small minority of foreign-born Latinx individuals (17–19 percent).

Table 2 presents results from mixed-effects logistic regression models examining factors associated with alcohol consumption, with odds ratios (ORs) and 95% confidence intervals (CIs) reported. All models account for within-individual clustering using a random intercept at the subject level (id) and adjust for age (including a quadratic term) and survey wave. The findings provide robust evidence of a selection effect, reflected in significantly lower odds of alcohol consumption among immigrant populations compared to their U.S.-born counterparts, with substantial variations by gender and ethnicity.

**Table 2:** Odds of alcohol consumption across nativity, gender, and ethnicity.

Variable		Model 1				Model 2				Model 3				Model 4			Model 5					Model 6			Model 7			
		OR		95% CI		OR		95% CI		OR		95% CI		OR		95% CI	OR			95% CI		OR		95% CI	OR		95% CI	
Immigrant		0.46		0.40–0.53		0.47		0.41–0.53																				
Female						0.52		0.46–0.59																				
Age		1.60		1.45–1.76		1.58		1.44–1.74		1.58		1.44–1.74		1.60		1.45–1.76	1.58			1.44–1.74		1.60		1.46–1.76	1.60		1.46–1.76	
Age-squared		0.99		0.98–0.99		0.99		0.98–0.99		0.99		0.98–0.99		0.99		0.98–0.99	0.99			0.98–0.99		0.99		0.98–0.99	0.99		0.98–0.99	
Wave 1 referent																												
Wave 3		1.10		0.85–1.43		1.15		0.89–1.48		1.14		0.88–1.47		1.11		0.86–1.43	1.16			0.90–1.50		1.12		0.87–1.45	1.11		0.86–1.44	
Wave 5		1.72		0.78–3.80		2.03		0.93–4.42		1.97		0.90–4.30		1.74		0.80–3.80	2.04			0.93–4.45		1.75		0.80–3.82	1.68		0.77–3.66	
Nativity*Gender (Nat. Male ref.)																												
Immigrant Male										0.54		0.45–0.65		0.60		0.50–0.73												
Immigrant Female										0.22		0.18–0.27		0.25		0.20–0.30												
Native-born Female										0.57		0.50–0.65		0.57		0.50–0.66												
Nativity*Gender*Ethnicity (Nat. Lat. Male ref.)																												
Asian Immigrant Male																	0.38			0.29–0.50		0.40		0.31–0.53	0.47		0.36–0.63	
Asian Immigrant Female																	0.20			0.15–0.26		0.21		0.16–0.28	0.25		0.18–0.34	
Variable	Model 1				Model 2				Model 3				Model 4					Model 5			Model 6					Model 7		
	OR		95% CI		OR		95% CI		OR		95% CI		OR		95% CI			OR	95% CI		OR		95% CI			OR		95% CI
Asian Native-born Male																		0.67	0.54–0.83		0.64		0.52–0.80			0.74		0.59–0.93
Asian Native-born Female																		0.44	0.35–0.56		0.42		0.33–0.53			0.48		0.37–0.61
Latinx Immigrant Male																		0.58	0.46–0.72		0.68		0.53–0.86			0.67		0.53–0.85
Latinx Immigrant Female																		0.20	0.15–0.26		0.23		0.18–0.30			0.23		0.17–0.30
Latinx Native-born Female																		0.53	0.45–0.63		0.54		0.46–0.63			0.53		0.45–0.62
Education									1.08		0.96–1.21		1.04		0.93–1.17			1.15	1.02–1.29		1.12		0.99–1.26			1.13		1.01–1.27
English use in intimate setting													1.30		1.15–1.47						1.38		1.22–1.57			1.40		1.23–1.59
Coethnic Friend Network (Asian)																										0.70		0.56–0.88
Coethnic Friend Network (Latino)																										1.12		0.96–1.31
Intercept	0.01		0.01–0.01		0.01		0.01–0.01		0.01		0.01–0.01		0.01		0.01–0.01			0.01	0.01–0.01		0.01		0.01–0.01			0.01		0.01–0.01
Intercept Variance (τ²)	1.17		0.95–1.45		1.06		0.85–1.33		1.05		0.84–1.32		1.03		0.82–1.30			1.04	0.83–1.31		1.01		0.80–1.27			0.99		0.78–1.26

Model 1 demonstrates a strong immigrant selection effect, with immigrants exhibiting 54 percent lower odds of alcohol consumption than the U.S.-born reference group (OR = 0.46, 95% CI: 0.40–0.53). Model 2 introduces gender as a covariate, indicating that females have significantly lower odds of consumption than males (OR = 0.52, 95% CI: 0.46–0.59). The immigrant protective effect remains substantial and largely, suggesting that the selection effect remains real independently of gender differences.

When the immigrant effect is disaggregated by gender in Model 3—using U.S.-born males as the reference—the selection effect remains pronounced. Immigrant males show significantly reduced odds of alcohol consumption (OR = 0.54, 95% CI: 0.45–0.65), and immigrant females exhibit an even stronger protective effect (OR = 0.22, 95% CI: 0.18–0.27). U.S.-born females also display lower odds than U.S.-born males (OR = 0.57, 95% CI: 0.50–0.65), though this effect is weaker than those among immigrants.

Model 4 incorporates additional covariates, including education and English use in intimate settings. The protective association for immigrant males (OR = 0.60, 95% CI: 0.50–0.73) and immigrant females (OR = 0.25, 95% CI: 0.20–0.30) persists after these adjustments. English use is positively associated with alcohol consumption (OR = 1.30, 95% CI: 1.15–1.47), suggesting a stronger role of acculturation.

Further stratification by ethnicity in Models 5 and 6—using U.S.-born Latinx males as the reference—reveals more nuanced patterns. The most substantial protective effects are observed among Asian immigrant women (Model 6 OR = 0.21, 95% CI: 0.16–0.28) and Latinx immigrant women (Model 6 OR = 0.23, 95% CI: 0.18–0.30), reflecting 75–80 percent lower odds relative to the reference group. Asian immigrant men also show markedly lower odds (OR = 0.40, 95% CI: 0.31–0.53), whereas Latinx immigrant men exhibit a more modest yet significant reduction (OR = 0.68, 95% CI: 0.53–0.86). Notably, the protective effect extends to the second generation, with U.S.-born Asian and Latinx women both demonstrating lower odds of alcohol consumption than U.S.-born Latinx males. In Model 7, the addition of co-ethnic friend network variables reveals different associations with the odds of alcohol consumption. Asian co-ethnic friend network shows protective effect (OR=0.70, 95% CI: 0.56–0.88), while Latino co-ethnic friend network variable shows statistically non-significant effect. The friendship network variables show mediating effects on the estimates on gender, nativity, and ethnicity cross-classifications, particularly among Asians.

**Fig. 1:**
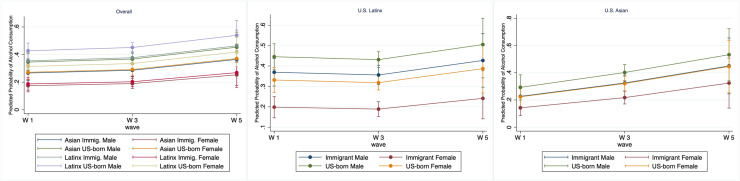
Three-panel comparison of alcohol consumption (Predicted) Probability: Overall (Left), Latinx (Center), and Asian (Right) subgroups by nativity and gender across survey waves. Immigrant females exhibit strongest protective patterns in both Latinx and Asian ethnicity.

The modeled findings were visually confirmed by margins plots showing predicted probabilities. A plot for the full sample (Fig. 1) showed persistently lower probabilities for immigrant groups across waves. When stratified by ethnicity, plots for the Asian and Latinx subsamples (Center Panel and Right Panel, respectively) reproduced the core results. Especially, Right Panel highlights the exceptionally strong protective effect for Latinx foreign-born women, who exhibited the lowest predicted probabilities of alcohol consumption.

In summary, these analyses consistently support a strong selection effect among immigrant groups, characterized by reduced alcohol consumption. This effect is most pronounced among immigrant women and Asian immigrant men and is partially mediated by acculturation. The persistence of these patterns across increasingly specified models underscores the robustness of the findings.

## 4 Discussion

This study employs longitudinal data to analyze alcohol consumption from adolescence to adulthood across Asian and Latinx Americans, with an objective to assess the diverse patterns of acculturative risks, examining the moderating roles of gender and nativity. This is the first study to utilize this extensive longitudinal source addressing the debate on behavioral assimilation post-migration (Ahmmad et al., 2023; Schwartz et al., 2010). The findings provide partial support for acculturative risk hypothesis but yield stronger evidence for the primacy of selection effects (Ahmmad et al., 2023; Eitle et al., 2009; Esser, 2004), a pattern that remains consistent across diverse ethnicities.

As hypothesized, immigrant adolescents reported significantly lower levels of alcohol consumption compared to their US-born peers. This finding was observed across descriptive statistics, cross-sectional analyses (available upon request), and longitudinal mixed-effects models. This result provides robust support for the well-established body of literature, both in the United States and internationally, which posits that immigrant populations arrive with protective behavioral profiles, regardless of age at arrival (Ahmmad and Adkins, 2021; Bacio et al., 2013; Eitle et al., 2009; Kopak, 2013; Peña et al., 2008).

Analysis provides partial support for the hypothesis that immigrants' health behavioral advantages erode with longer US residence, potentially driven by host society contact (e.g., English acquisition) (Bacio et al., 2013; Eitle et al., 2009; Kopak, 2013; Lui and Zamboanga, 2018; Peña et al., 2008; Zemore, 2007). Mixed-effects models show a slight upward alcohol consumption trajectory for some immigrant subgroups, though not all. Notably, data do not support full convergence to high-risk groups like (Latinx) US-born males, even across multi-decades in their life course. English use had a significant, sizable effect on increased consumption, particularly among Latinx individuals, suggesting its role as a mechanism for negative acculturation (Zemore, 2007). The stronger effect in Latinx versus Asian subgroups may stem from differing linguistic contexts; wider Spanish usage may make English use in intimate setting a stronger acculturation marker, potentially loosening origin community protective effects (Zemore, 2007). However, English use did not fully mediate nativity, gender and alcohol consumption relationships. The protective role of co-ethnic friend networks also diverged for different ethnicities. A higher proportion of co-ethnic friends was protective for Asian Americans (Ahmmad and Adkins, 2021). For Latino Americans, however, no consistent pattern emerged, supporting prior research and underscoring how social capital might operate differently across immigrant communities (Eitle et al., 2009).

**Fig. A.1:**
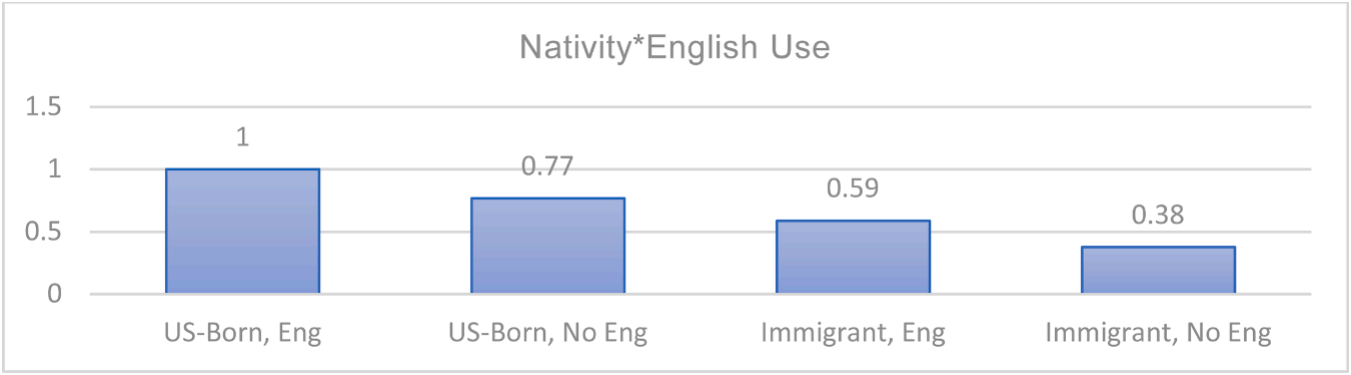
Nativity and English Use Interactions from the Fully Adjusted Model *Note.* U.S.-born individuals who use English in personal conversation had the highest odds of binge drinking, followed by U.S.-born individuals who do not use English in personal conversation, then immigrants who use English, and finally immigrants who do not use English. All results are statistically significant.

**Fig. A.2:**
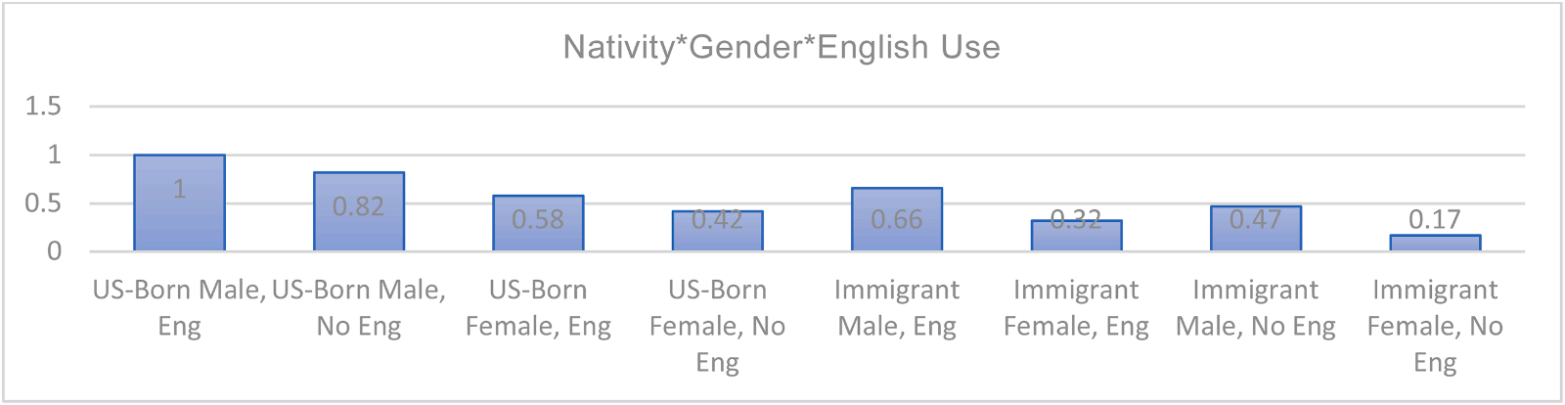
Nativity, Gender, and English Use Interactions from the Fully Adjusted Model *Note.* All results are statistically significant except for U.S.-born male using no English in personal conversation. Notably, gender remains a strong selection indicator in the interaction model. For instance, immigrant males who use English in personal conversation had substantially higher odds of binge drinking (0.66) than female immigrant counterparts (0.32).

The analysis lends extensive supports to the hypothesis that immigrant acculturation can be selective (Ahmmad et al., 2023; Lui and Zamboanga, 2018; Wahl and Eitle, 2010; Yeh et al., 2009). Outcomes are shaped by differential socioeconomic selection and varying cultural backgrounds across ethnicities. Specifically, pre-migration gender norms appear to exert an enduring influence on subsequent behavioral patterns (Kamimura et al., 2018). This assumption is strongly substantiated by the markedly flatter growth trajectories in alcohol consumption observed among immigrant girls from both Asian and Latinx backgrounds. A particularly notable finding emerged among Latinx immigrant girls. Despite perceptions of Latinx cultures as more permissive toward alcohol (Hernández-Vásquez et al., 2022), this subgroup exhibited the flattest growth, indicating a potentially strong selective effect. While immigrant males also displayed evidence of selective retention—as their consumption rates did not fully converge with US-born peers over time—the protective magnitude of this effect was significantly weaker than that observed among females. Furthermore, sensitivity analyses (Appendix Fig. A.1, Fig. A.2) show that English use has interactive and differential effects across nativity and gender, reinforcing gender as a selection indicator. A sizable difference in the odds of binge drinking was found between male and female immigrants who use English in personal conversation (OR=0.66 vs. 0.32). This pattern underscores that gender remains a fundamental axis of differentiation in the selection and maintenance of health behaviors among contemporary US immigrant groups (Ahmmad et al., 2023; Wahl and Eitle, 2010).

Although higher educational attainment, as a marker of greater integration, can be expected to predict higher odds of alcohol consumption, findings from this study offer no significant support for this. Previous literature, however, suggests that the education-alcohol relationship is ethnicity specific and was found to have an inverted U-shaped relationship between education and alcohol use among different immigrant groups (Szaflarski et. al., 2011). The findings in this study align with the non-significant findings in previous literature focused on Asians (Ahmmad and Adkins, 2021) and Latino immigrants (Levitt et. al., 2019). Future research should investigate the interactive effects of multiple levels of education and English proficiency, as these interactive factors may shape health behaviors synergistically rather than independently (see Appendix Table A.1).

Findings from this study underscore the necessity of longitudinal research designs to accurately study immigrant acculturation. Unlike studies reliant on retrospective self-reporting—which may misattribute outcomes to progressive acculturative markers like English use or local social networks—longitudinal data captures nuanced developmental trajectories and temporal dynamics that cross-sectional studies may fail to observe. This research leverages a unique adolescent cohort, offering distinct advantages over adult samples for examining acculturation processes. Adolescents are less affected by professional motivations for language use, recall bias regarding migration history, or strong transnational ties, allowing clearer isolation of acculturative change. As this group underwent formative development within U.S. educational and social systems, they represent an informative population for studying accelerated acculturation and potential behavioral shifts due to “Americanization” (Cobb et al., 2021).

While the sample consists of school-enrolled youth, potentially limiting generalizability to non-schooled peers, secondary evidence suggests that structural barriers—such as legal status and institutional access—likely exert dampening influences on acculturation efforts. Thus, the absence of non-schooled individuals may not substantially bias the observed patterns.

A key limitation of this study is the aggregation of diverse origin groups into broad pan-ethnic categories, such as “Asian” (predominantly East Asian in this sample). This approach obscures meaningful heterogeneity in selection mechanisms, cultural backgrounds, and pathways of integration across subgroups, as highlighted in previous literature (Ahmmad and Adkins, 2021). Although our findings indicate that substance use disparities by pan-ethnicity are notably consistent and do not vary significantly by gender, future research should strive to incorporate more nuanced geographic, cultural, and national-origin identifiers where possible.

Data for the most recent Add Health Wave were collected between 2016 and 2018. While not contemporaneous, the data age does not limit the study’s aims to validate acculturation theoretical frameworks using health behaviors with varying cultural sensitivity (e.g., smoking versus alcohol use). This study offers more nuanced insights by including Latinx ethnicity and focusing on binge drinking—a distinct behavioral marker beyond the any alcohol use assessed in prior work (Ahmmad and Adkins, 2021). Given the relative stability of observed divergences over time (Fig. 1), more recent observations are unlikely to yield substantially different results.

Future studies should expand the analytical focus to lifestyle factors—such as physical activity—and health indicators like self-rated health, to provide a more comprehensive understanding of immigrant health adaptation. Additionally, extending this research to understudied immigrant populations, including White, Black, African, and Middle Eastern immigrants, would help validate and generalize these findings across a broader demographic spectrum (Read et al., 2020).

## Compliance of ethical standards

This study adhered to ethical standards set by the University of Vermont's Institutional Review Board (IRB). As the study used de-identified secondary data, it was exempt from full IRB review.

## Data availability

The Add Health data are available from the Carolina Population Center at the University of North Carolina at Chapel Hill. Public-use data are available at https://addhealth.cpc.unc.edu/data/public-use-data-sets/. Access to the restricted data used in this study is subject to a data use agreement.

## Funding

There is no funding to declare.

## CRediT authorship contribution statement

**Zobayer Ahmmad:** Writing – review & editing, Writing – original draft, Visualization, Validation, Software, Methodology, Formal analysis, Data curation, Conceptualization.

## Declaration of competing interest

I, Zobayer Ahmmad, the sole author of this manuscript, declare that I have no relevant financial or non-financial interests to disclose.

## Figures and Tables

**Table A.1:** Multicollinearity diagnostics between English use and educational attainment variables.

**Diagnostic Test**	**Value**	**p-value**	**Interpretation**

Pearson Chi-Square (χ2)	275.85	p < 0.001	Significant association; variables are not independent.
Cramer's V	0.17	-	Weak relationship; well below the 0.70 redundancy threshold.
VIF for Education	1.20	-	Negligible inflation of variance; coefficient stability is high.
VIF for English Use	1.28	-	Negligible inflation of variance; coefficient stability is high.
Tolerance (1/VIF) for Education	0.83	-	High unique variance (83%); no collinearity concern.
Tolerance (1/VIF) for English Use	0.78	-	High unique variance (78%); no collinearity concern.
